# Modulation of TLR2, TLR4, TLR5, NOD1 and NOD2 receptor gene expressions and their downstream signaling molecules following thermal stress in the Indian major carp catla (*Catla catla*)

**DOI:** 10.1007/s13205-015-0306-5

**Published:** 2015-05-16

**Authors:** Madhubanti Basu, Mahismita Paichha, Banikalyan Swain, Saswati S. Lenka, Samarpal Singh, Rina Chakrabarti, Mrinal Samanta

**Affiliations:** Fish Health Management Division, Central Institute of Freshwater Aquaculture (CIFA), Kausalyaganga, Bhubaneswar, 751002 Orissa India; Aqua Research Lab, Department of Zoology, University of Delhi, Delhi, 110007 India

**Keywords:** *Catla catla*, Water temperature, Thermal stress, TLR, NOD

## Abstract

Toll-like receptors (TLRs) and nucleotide binding and oligomerization domain (NOD) receptors are pattern recognition receptors (PRRs) that recognize pathogen-associated molecular patterns (PAMPs) and play crucial role in innate immunity. In addition to PAMPs, PRRs recognize endogenous molecules released from damaged tissue or dead cells [damage-associated molecular patterns (DAMPs)] and activate signaling cascades to induce inflammatory processes. In the aquatic environment, large variation in seasonal and diurnal water temperature causes heat and cold stresses in fish, resulting in tissue injury and mortality of fish. In the Indian subcontinent, catla (*Catla catla*) is an economically important freshwater fish species and is prone to thermal stresses. To investigate the response of pattern recognition receptors in thermal stress, we analyzed TLRs (TLR2, TLR4 and TLR5) and NOD (NOD1 and NOD2) receptors gene expression in catla following heat and cold stress. Analysis of tissue samples (gill, liver, kidney and blood) of the thermal stressed and control fish by quantitative real-time PCR (qRT-PCR) assay revealed significant (*p* < 0.05) induction of TLR2, TLR4 and NOD2 gene expression in majority of the tested tissues of the treated fish as compared to the control. The expression of TLR5 and NOD1 gene was also induced in the heat and cold stressed fish, but mostly restricted in the blood. The downstream signaling molecule of TLR and NOD signaling pathway viz., MyD88 (myeloid differentiation primary response gene 88) and RICK (receptor interacting serine-threonine protein kinase-2) was also induced in the thermal stressed fish suggesting the engagement of TLR and NOD signaling pathway during thermal stress.

## Introduction

The success of aquaculture depends on providing an optimum and congenial environment to fish which subsequently helps to achieve their higher survival rate and growth (Boyd and Tucker 1998). The health status of aquatic animals is uniquely influenced by their immediate surroundings viz., pH, salinity, temperature, ambient light intensity, presence of contaminants, dissolved oxygen concentration etc. (Tort 2011). Among these, temperature is one the most important abiotic factors that plays a critical role in the life of poikilothermic animals like fish (Fry 1971; Brett 1971; Stewart et al. 2002). Various fish species differ in requirement of their optimal temperature (Sharma et al. 2014). Beyond that limit, fishes experience the thermal stress resulting in tissue injury and are prone to be infected by opportunistic pathogens (Das et al. 2004; Gordon 2005; Dalvi et al. 2009). To defend against pathogenic invasion, fish primarily depend upon non-specific or innate immunity contributed by pattern recognition receptors (PRRs) like toll-like receptors (TLRs) and nucleotide binding and oligomerization domain (NOD) receptors. In addition to the PAMPs (pathogen-associated molecular patterns) recognition, TLRs sense DAMPs (damage-associated molecular patterns) which are endogenous host molecules viz., fibronectin (Okamura et al. 2001), heparin sulfate (Johnson et al. 2002), biglycan (Schaefer et al. 2005), fibrinogen (Smiley et al. 2001), oligosaccharides of hyaluronan breakdown products (Jiang et al. 2005; Taylor et al. 2004, 2007), heat shock proteins (Yu et al. 2010), high mobility group box1 (HMGB1) (Tang et al. 2011), tenascin-C (Midwood et al. 2009), cardiac myosin (Zhang et al. 2009), S100 proteins, thioredoxin-interacting protein (TXNIP) (Yu et al. 2010) etc. Physiologically, these endogenous ligands are localized under different cellular compartment, but under stress they are either released passively from the injured tissues/dying cells or actively secreted by activated cells via non-conventional lysosomal route (Pollanen et al. 2009). Endogenous TLR ligands act as alarmins and may serve as early warning signals to innate and adaptive immunity (Matzinger 2002; Seong and Matzinger 2004). Recognition of DAMPs by PRRs activates signaling cascade resulting in the induction of cytokines, recruitment of more immune cells and repair of damaged tissue (Medzhitov 2008). In addition to TLRs, NOD-like receptors (NLRs) have also been shown to respond to both microbial components (Franchi et al. 2012) and endogenous ligands derived from tissue/cellular injuries (Ting et al. 2008; Tschopp and Schroder 2010; Krishnaswamy et al. 2013; Monie 2013).

The global climate is rapidly changing, resulting in significant shift in water temperatures and stresses in various fish species (Jain and Kumar 2012). The variation in water temperature has been shown to modulate the expression of TLR gene transcripts in zebrafish (*Danio rerio*) (Sundaram et al. 2012). In India, among various freshwater fish species, catla (*Catla catla*) is one of the most commercially important and highly favored fish in the farming industry. Therefore, this work was undertaken to investigate the response of TLRs and NOD receptors in catla during thermal stresses.

## Materials and methods

### Fish

Catla fry (0.676 ± 0.026 g) was obtained from a local fish farm and was stocked in 50 L glass aquaria in the wet laboratory. Before the start of the experiment, acclimatization was carried out for 3-weeks at 25 °C to avoid the handling stress. The fish was fed twice a day with laboratory prepared feed (40 % protein) at the rate of 5 % of their body weight. Aeration was constant to maintain high oxygen level (6.21–7.14 mg/l) and the continuous mixing of water throughout the study period.

### Thermal stress and sampling

Fish were randomly distributed in 12 glass aquaria each containing 40 fish. Each aquarium was connected with a filtration unit and a cooling/heating unit. This helped to maintain desirable temperature in the aquarium. The used water from fish culture unit was first circulated into the filtration unit, then to the cooling or heating unit, and finally into the fish culture unit. The ambient temperature was 25 ± 2 °C during this period. Fish acclimatized at 25 °C were exposed to six different temperature viz. 10, 15, 20, 25, 30 and 35 °C. Two replicates were used for each temperature. The 25 °C temperature was considered as ambient temperature (control). Two groups were maintained above and three groups were below the control temperature. Each experimental temperature was achieved with change of temperature at 1 °C/12 h starting from acclimatization temperature of 25 °C. In this way, the treated aquaria attained 20 and 30 °C after 2.5 days, and 10 °C after 7.5 days.

For sampling, fish were collected from each tank (*n* = 10) after 12 h of achieving the assigned temperature to study the immediate effect of stress after exposure. Then again fish were collected after 7 days to study the effect of chronic stress. Total two samplings were conducted in all treatments, except at 10 °C because fish died before second sampling. Fish were taken out from experimental tank, anesthetized with MS-222 (Sigma, USA) following which gill, liver, kidney and blood were collected separately in TRIzol reagent for RNA extraction and further study.

### RNA isolation, cDNA synthesis and quantitative real-time PCR analysis

Total RNA was extracted from TRIzol reagent-treated sample following the manufacturer’s protocol (Invitrogen, USA). The concentration of RNA was measured by UV-spectrophotometer (Biophotometer Plus, Eppendorf, Germany), and the quality was assessed by observing the intensity of 28 and 18S rRNA (ribosomal RNA) band in 1 % agarose gel. To synthesize the first strand cDNA (complementary DNA), 1 µg of total RNA was first treated with 1 unit of DNase I (MBI, Fermentas, USA), and was reverse transcribed with oligo-dT primer and RevertAid 1st strand cDNA synthesis kit (MBI, Fermentas, USA). The PCR-amplification of β-actin gene was carried out for the confirmation of cDNA synthesis.

To study the basal expression of innate immune genes (TLR2, TLR4, TLR5, NOD1 and NOD2) in gill, liver, kidney and blood of catla fry, and their in vivo modulation following variations in water temperatures, quantitative real-time PCR (qRT-PCR) was employed. The qRT-PCR was performed in LightCycler^®^ 480 II real-time PCR detection system (Roche, Germany), and in a 10 µl reaction volume the following reagents were added: cDNA-1 µl, FW and RV primer (2.5 µM each; Table 1) 0.25 µl each, 2X LightCycler^®^ 480 SYBR Green I master mix (Roche, Germany) 5 µl and PCR grade water 3.5 µl. PCR amplifications were performed in triplicate wells under the following conditions: initial denaturation at 95 °C for 10 min followed by 45 cycles of 94 °C for 10 s, 51 °C (MyD88)/55 °C (TLR4)/56 °C (NOD1)/58 °C (TLR2, TLR5, RICK and β-actin)/60 °C (NOD2) for 10 s and 72 °C for 10 s. For negative control, qRT-PCR reaction without cDNA was considered. To determine PCR efficiencies, qRT-PCR with serial dilutions of cDNA was carried out. The efficiencies were ~100 %, that allowed the use of 2^−ΔΔCT^ method to calculate relative gene expression of the target genes with that of reference gene, β-actin. In a 2 % agarose gel, 8 µl of the real-time PCR products were loaded to verify the specificity of the product size. The relative expression ratios were obtained by normalizing expression of the target gene, as determined by mean crossing point (cp) deviation, by that of reference gene, β-actin following 2^−ΔΔCT^ method (Livak and Schmittgen 2001). The data obtained from qRT-PCR analysis were expressed as mean of two experiments ± standard error (SE), and the significant difference between control and treated groups at each time point was determined by the Student’s *t* test using Microsoft Excel 2010 with *p* < 0.05 as significance level.

**Table 1:** Primers used for quantitative real-time PCR (qRT-PCR) analysis

Target gene	Primer name	Sequence (5′→3′)	Annealing temp (°C)	Amplicon size (bp)	GenBank ID
TLR2	TLR2 FW	GACGGTCATGGATGGTTCTTCTTTA	58	131	HQ293022
	TLR2 RV	CAAGATTGCGTATGTAGGCCGTATG			
TLR4	TLR4 FW	ATGATGGAGCGCAATGCCAA	55	140	GU248418
	TLR4 RV	ATGTTACTCAAAGGGTCTCTGCTCC			
TLR5	TLR5 FW	CAGGGTAAACATTTCACGCTTCT	58	162	GU230763
	TLR5 RV	ACGCTTTGCCATGGGAACTTT			
NOD1	NOD1 FW	GTTGGTGGGAAATACCTTGCC	56	217	KC542884
	NOD1 RV	TGCTTTCGCCAGACTTCTTCC			
NOD2	NOD2 FW	GGCGGGACAGGACGTTTCTCC	60	261	KC542885
	NOD2 RV	GCGGCAACTGAAGGGGAATA			
MyD88	MyD88 FW	CTTCCAGTTTGTGCATGAGA	51	146	JN247432
	MyD88 RV	CCATCCTCTTGCACCTTTTT			
RICK	RICK FW	GGCGCCAGCTCTCTATCACTAA	58	186	KC542886
	RICK RV	CCTCTTCAAATGGTATCCGTCTT			
β-actin	β-actin FW	AGACCACCTTCAACTCCATCATG	58	200	EU184877
	β-actin RV	TCCGATCCAGACAGAGTATTTACGC			

## Results and discussion

### Tissue specific expression of innate immune genes

**Fig. 1:**
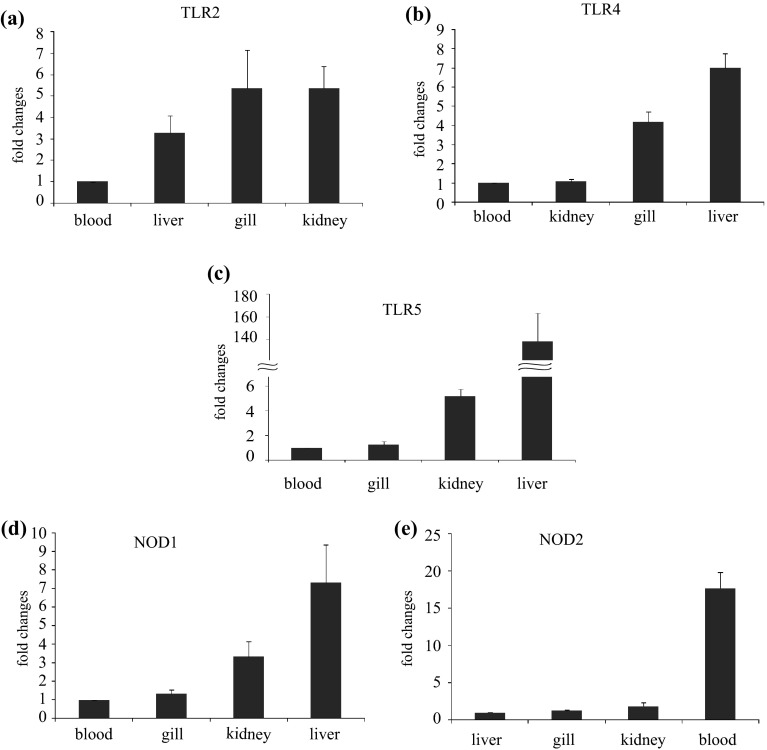
Tissue specific basal expression of TLR2, TLR4, TLR5, NOD1 and NOD2 gene. Total RNA was extracted from gill, liver, kidney and blood of catla fry. Quantitative real-time PCR (qRT-PCR) was carried out to analyze TLR2, TLR4, TLR5, NOD1 and NOD2 gene expression among the tissues. Expressions of these gene transcripts were represented as a ratio relative to β-actin (internal control) levels in the same samples. The tissue which showed lowest expression of respective gene was chosen as calibrator (1), and the relative expression of TLR2, TLR4, TLR5, NOD1 and NOD2 gene in other tissues was represented as fold changes from the calibrator. The results were expressed as mean ± standard error (*bars* in the graph) from ten fish (*n* = 10). **a** TLR2, **b** TLR4, **c** TLR5, **d** NOD1 and **e** NOD2 gene expression

The data of qRT-PCR revealed wide expression of TLR2, TLR4, TLR5, NOD1 and NOD2 genes across the tested organs/tissues, but the magnitude of their expression varied. Lowest expression of TLR2, TLR4 and TLR5 was observed in blood. As compared to the blood, TLR2 gene expression in gill and kidney was ~5.5 fold (Fig. 1a), and TLR4 and TLR5 in liver was ~7 fold (Fig. 1b) and ~150 fold (Fig. 1c), respectively. Among the NOD receptors, least expression of NOD1 was detected in blood and the highest (~7 fold) was in liver (Fig. 1d). In contrast, NOD2 expression was lowest in liver and highest (~20 fold) in blood (Fig. 1e). The expression of TLR2, TLR5, NOD1 and NOD2 gene in the embryonic developmental stages and in various organs/tissues was previously been reported in *Labeo rohita* (Samanta et al. 2012; Swain et al. 2012, 2013a, b) and *Cirrhinus mrigala* (Basu et al. 2012a, b, 2013). Catla is a closely related fish to rohu and mrigal. Therefore, the expression of TLR and NOD genes in gill, liver, kidney and blood was expected. The constitutive expression of TLR and NOD genes may indicate their availability as innate immune receptor in the early developmental stages of catla.

### Modulation of TLR and NOD gene expression

We monitored immediate (12 h post-exposure) and late (7 days post-exposure) response of TLR2, TLR4, TLR5, NOD1 and NOD2 receptors in catla fry by analyzing the tissue samples (gill, liver, kidney and blood) of control fish (fish maintained at 25 °C), cold stressed fish (exposed to 10, 15 and 20 °C) and heat stressed fish (exposed to 30 and 35 °C) through quantitative real-time PCR (qRT-PCR) assay. At 7 days post-exposure, all catla fry remained alive at 15, 20, 30 and 35 °C, but at 10 °C, all fish died.

### Toll-like receptor-2

**Fig. 2:**
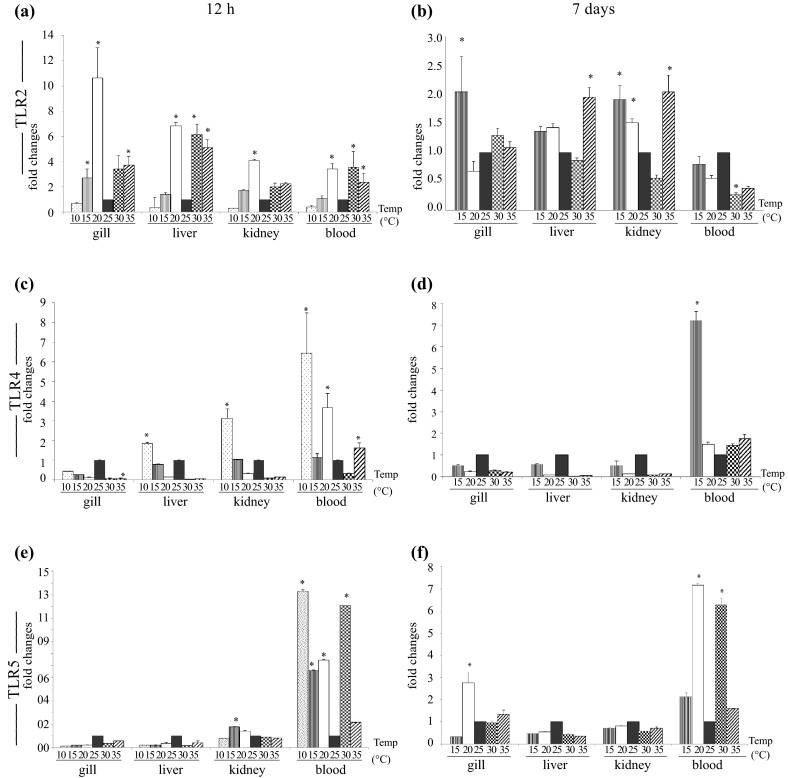
TLR2, TLR4 and TLR5 gene expression in thermal stress. Total RNA was extracted from gill, liver, kidney, and blood from the control and treated fish, at 12 h and 7 days post-exposure, and quantitative real-time PCR was conducted to analyze TLR2, TLR4 and TLR5 gene expression keeping β-actin as housekeeping control gene. The results were calculated as mean ± standard error (*bars* in the graph) and were shown as fold changes compared to control. Significant difference (*p* < 0.05) between control and treated fish group was indicated with *asterisks*. TLR2 gene expression at 12 h (**a**) and 7 days (**b**); TLR4 gene expression at 12 h (**c**) and 7 days (**d**) and TLR5 gene expression at 12 h (**e**) and 7 days (**f**) post-treatment

In response to the early (12 h) thermal stress, TLR2 gene expression was significantly (*p* < 0.05) up-regulated in gill, liver, kidney and blood of the treated fish as compared to control (Fig. 2a). In gill, TLR2 expression was highest at 20 °C (~10 fold) and it declined gradually with further lowering of temperature. A similar trend of TLR2 expression was also observed in liver, kidney and blood at 20 and 15 °C. At 10 °C, TLR2 expression was found to be suppressed in all tested tissues. Due to heat stress, TLR2 induction in gill, liver, kidney and blood was also increased at 30 and 35 °C. Among the organs, maximum induction of TLR2 was observed in liver at 30 °C (~6 fold) followed by gill and blood. Fish exposed at 35 °C expressed higher TLR2 in all tested tissues than the control fish maintained at 25 °C. During late thermal stress (7 days), the pattern of TLR2 gene expression in gill, liver and kidney was almost similar as observed in early (12 h) thermal stress but the magnitude of the response in terms of fold change was different (Fig. 2b). In contrast to other tissues, TLR2 gene expression in blood was down-regulated in the treated fish group as compared to control.

### Toll-like receptor-4

The organ/tissue specific modulation of TLR4 was detected in thermal stressed fish at 12 h post-exposure as compared to control fish (Fig. 2c). In gill, TLR4 expression was down-regulated at all experimental temperatures as compared to control. In liver, except at 10 °C, a similar trend of TLR4 gene expression was also observed. In kidney, a significant induction of TLR4 was noted at 10 °C, and at higher temperatures (30 and 35 °C) it was down-regulated. In blood, highest induction of TLR4 gene expression was observed at 10 °C as compared to the control fish. At 7 days post-thermal stress (Fig. 2d), TLR4 gene expression in gill, liver and kidney of cold and heat stressed fish showed down-regulation. However, in blood there was marginal increase in TLR gene expression at 20 °C, which reached to its peak at 15 °C (~7 fold). There was moderate increase in TLR4 gene expression at 30 and 35 °C (~2 fold) as compared to the control fish.

### Toll-like receptor-5

The induction of TLR5 gene expression in thermal stressed fish was also tissue specific as compared to the control fish. At 12 h post-exposure, TLR5 was down-regulated in gill and liver at all experimental temperatures (Fig. 2e). In kidney, TLR5 expression showed a marginal increase at 15 °C (~1.7 fold) and 20 °C (~1.4 fold) as compared to control, but at other temperatures it remained almost unchanged. In blood, the pattern of TLR5 gene expression was strikingly different from other tested tissues. As compared to the control, most significant (*p* < 0.05) induction of TLR5 was observed in blood during cold stress at 10 °C (~14 fold) and heat stress at 30 °C (~13 fold). At 7 days post-thermal stress, except in gill, the trend of TLR5 gene expression in liver, kidney and blood of treated fish group was almost similar to 12 h post-thermal stress (Fig. 2f). In the treated fish gill, TLR5 gene expression was up-regulated at 20 °C as compared to the control fish.

The TLR2 and TLR4 are reported to be responsible for recognition of heat shock proteins (Hsp60, Hsp90 and Gp96), HMGB1 (Tsan and Gao 2004) and hyaluronan-induced inflammatory response (Jiang et al. 2005; Noble and Jiang 2006). Similarly, in catla, the activation of TLR2 and TLR4 is likely to be mediated through Hsp during heat stress and HMGB1 released from the necrotic cells (Tsung et al. 2005). In addition to these, other endogenous TLR ligands released from damaged tissue and cells may activate TLRs. Recognition of endogenous ligands by TLR5 was previously been reported in rheumatoid arthritis (Chamberlain et al. 2012). In catla, significant induction of TLR5 gene expression in blood suggests tissue injury resulting in the release of endogenous TLR5 ligands. However, further works are necessary to draw any conclusion in this regard.

### Nucleotide binding and oligomerization domain (NOD)-1

**Fig. 3:**
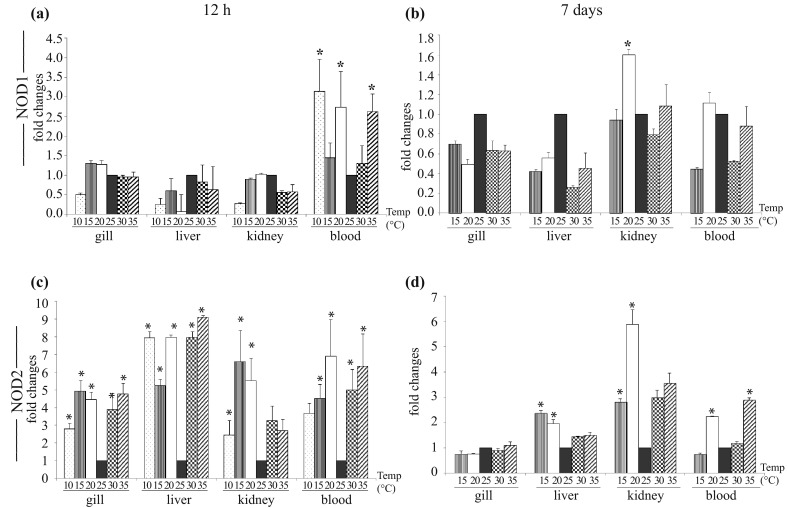
NOD1 and NOD2 gene expression in thermal stress. Total RNA was extracted from gill, liver, kidney, and blood from the control and treated fish, at 12 h and 7 days post-exposure, and quantitative real-time PCR was conducted to analyze NOD1 and NOD2 gene expression keeping β-actin as housekeeping control gene. The results were calculated as mean ± standard error (*bars* in the graph) and were shown as fold changes compared to control. Significant difference (*p* < 0.05) between control and treated fish group was indicated with *asterisks*. NOD1 gene expression at 12 h (**a**) and 7 days (**b**); NOD2 gene expression at 12 h (**c**) and 7 days (**d**)

We next analyzed NOD1 gene expression in thermal stressed and control fish at 12 h post-exposure (Fig. 3a). Among all tested tissues, the most significant induction (*p* < 0.05) of NOD1 was observed in blood. During cold stress, NOD1 expression in blood was ~2.7 fold at 20 °C, and it reached the peak (~3 fold) at 10 °C. In heat stress, there was gradual increase in NOD1 expression at 30 °C (~1.3 fold) and 35 °C (~2.6 fold). In gill, NOD1 gene expression was observed to be slightly induced or remained unchanged. In liver and kidney, down-regulation of NOD1 was observed in response to cold as well as heat shock. As shown in Fig. 3b, NOD1 gene expression in cold and heat exposed fish gill and liver remained down-regulated at all tested temperatures after 7 days post-exposure. However, in kidney it was up-regulated (~1.6 fold) only at 20 °C as compared to the control fish. In blood, NOD1 expression remained down-regulated at 15, 30 and 35 °C, but a marginal up-regulation was observed at 20 °C in the treated fish group.

### Nucleotide binding and oligomerization domain (NOD)-2

The effect of thermal stress (both cold and heat) on the catla NOD2 gene expression was clearly different from other tested PRRs. As compared to the control (25 °C), NOD2 was significantly (*p* < 0.05) up-regulated during thermal stress in all tested tissues/organs at 12 h post-exposure (Fig. 3c). With the advancement of temperature from 25 °C, there was gradual increase in NOD2 gene expression in all tested organs/tissues, except in kidney. Among the tissues, highest induction of NOD2 was observed in liver (~9 fold). In gill and kidney, maximum induction of NOD2 was observed at 15 °C (5–7 fold), and it gradually decreased with the lowering of temperature to 10 °C. In blood, we noticed steady increase in NOD2 expression following cold and heat stress. At 7 days post-exposure, liver, kidney and blood of treated fish group also revealed enhanced NOD2 gene expression (Fig. 3d). Among the tissues, maximum induction of NOD2 was in kidney: at 20 °C it was ~6 fold, at 15 °C ~2.8 fold, at 35 °C ~3.5 fold and at 30 °C it was ~3 fold as compared to the control. In liver, highest expression of NOD2 was at 15 °C (~2.4 fold) and in blood at 35 °C (~2.9 fold).

In addition to PAMPs recognition, the response of NOD receptors in recognizing endogenous ligands (DAMPs) was reported during tissue injury (Ting et al. 2008; Tschopp and Schroder 2010). In fish, the response of NOD receptors in PAMPs recognition and innate immunity was previously been reported in rohu, catla and mrigal (Swain et al. 2012, 2013a, b). In thermal stress, the activation of NOD1 and NOD2 gene in some of the tissues of catla supports the previous observation of DAMPs recognition by NOD receptors, and warrant further study in this regard.

### Modulation of TLR and NOD receptor associated downstream signaling molecules

#### Myeloid differentiation primary response gene 88

**Fig. 4:**
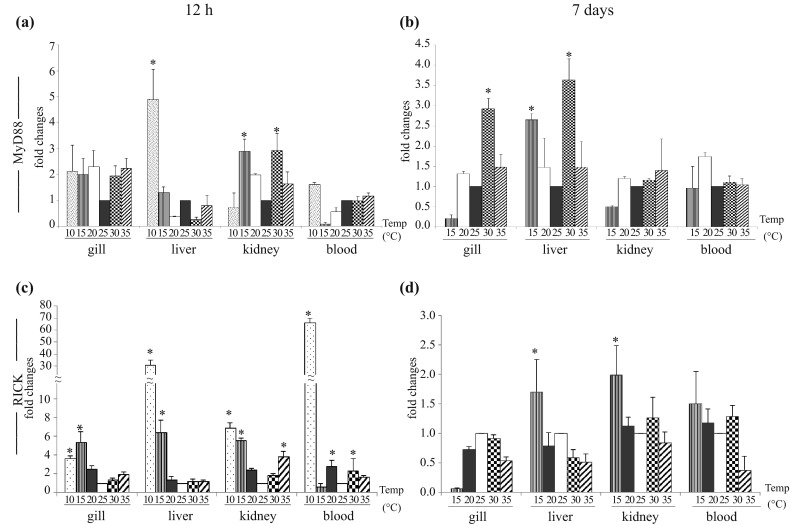
MyD88 and RICK gene expression in thermal stress. Total RNA was extracted from gill, liver, kidney and blood from the control and treated fish at 12 h and 7 days post-exposure, and quantitative real-time PCR was conducted to analyze MyD88 and RICK gene expression keeping β-actin as housekeeping control gene. The results were calculated as mean ± standard error (*bars* in the graph) and were shown as fold changes compared to control. Significant difference (*p* < 0.05) between control and treated fish group was indicated with *asterisks*. MyD88 gene expression at 12 h (**a**) and 7 days (**b**); RICK gene expression at 12 h (**c**) and 7 days (**d**)

MyD88 is the downstream adaptor molecule in TLR2, TLR4 and TLR5-signaling pathway. At 12 h post-cold stress and heat stress, MyD88 gene expression in the treated fish gill increased ~2 fold as compared to the control. In the treated fish liver, there was ~5 fold increase in MyD88 expression at 10 °C. At 15 and 35 °C, MyD88 expression was almost equal to the control and at 20° and 30 °C, it was down-regulated. In kidney, except at 10 °C, there was inductive expression of MyD88 gene (2–3 fold), and it reached maximum of ~3 fold at 15 and 30 °C. In blood, there was ~2 fold increase in MyD88 expression only at 10 °C (Fig. 4a). At 7 days post-treatment, MyD88 gene expression increased significantly in gill and liver of the treated fish group as compared to control, and it reached its peak (~3 fold) at 30 °C. In kidney, MyD88 remained almost unchanged at 20, 30 and 35 °C but it was down-regulated at 15 °C. In blood, there was up-regulation of MyD88 at 20 °C, and at other temperatures it remained almost unchanged (Fig. 4b).

In MyD88-dependent TLR-signaling pathway, recognition of PAMP or DAMPs by TLR2, TLR4 and TLR5 leads to the activation of downstream adaptor molecule “MyD88” resulting in NF-κB phosphorylation, and induction of cytokines gene expression (Akira 2009). In this study, up-regulation of either TLR2/TLR4/TLR5 genes expression in some tissues correlated with MyD88 gene expression during cold and heat shock, suggesting the activation of MyD88-dependent TLR-signaling pathway. Previously, MyD88 activation in PAMPs mediated TLR2, TLR4 and TLR5 signaling resulted in the induction of cytokines in rohu (Samanta et al. 2012) and mrigal (Basu et al. 2012a, b, 2013). Catla, a member of the Indian major carps (IMC), is closely related to rohu and mrigal under the same family of *Cyprinidae*. Therefore, activation of MyD88 in DAMP mediated TLR signaling during cold and heat stresses may follow a similar pathway of NF-κB phosphorylation and cytokine gene expression.

#### Receptor interacting serine-threonine protein kinase-2 (RICK)

In NOD1 and NOD2 signaling pathway, RICK functions as downstream adaptor molecule. We investigated RICK gene expression in gill, liver, kidney and blood of control and thermal stressed fish through qRT-PCR. As shown in Fig. 4c, there was significant induction of RICK gene expression at 12 h post-treatment in all tested tissues. Due to cold stress, highest induction of RICK was observed at 10 °C in blood, followed by liver, kidney and gill in the treated fish group as compared to the control. During heat stress all other tissues except liver revealed marked increase in RICK gene expression. At 7 days post-treatment, RICK gene expression in liver, kidney and blood of the treated fish group followed almost similar pattern as observed in 12 h post-treatment. However, the magnitude of RICK induction at 7 days was much lower than 12 h (Fig. 4c).

NOD1 and NOD2 are cytoplasmic sensors of PAMP/DAMPs and they transmit downstream signaling through RICK. In rohu, catla and mrigal, activation of NOD1, NOD2 and RICK gene expression was previously been reported following PAMPs (iE-DAP, LPS and poly I:C) stimulation and bacterial infection (Swain et al. 2012, 2013a, b). In cold and heat stresses, we also noted significant up-regulation of NOD1 and NOD2 and RICK gene expression in various tissues, which may suggest the activation of NOD signaling pathway by the endogenous ligands during thermal stress in catla.

## Conclusion

This article demonstrates TLR2, TLR4, TLR5, NOD1 and NOD2 gene expression in catla fish during the early developmental stages, and this is the first report. The inductive expression of TLR and NOD receptor genes along with their downstream molecules MyD88 and RICK, respectively, suggests the release of DAMPs during thermal stress in fish. The data in this study may help in investigating the greater role of TLR and NOD receptors in repairing the damage tissues and pathology of fish.
